# Use of Partition Coefficients in a Hexane–Acetonitrile System in the GC–MS Analysis of Polyaromatic Hydrocarbons in the Example of Delayed Coking Gas Oils

**DOI:** 10.1021/acsomega.1c00691

**Published:** 2021-03-31

**Authors:** Ignaty Efimov, Vladimir Glebovich Povarov, Viacheslav A. Rudko

**Affiliations:** Saint Petersburg Mining University, St. Petersburg 199106, Russia

## Abstract

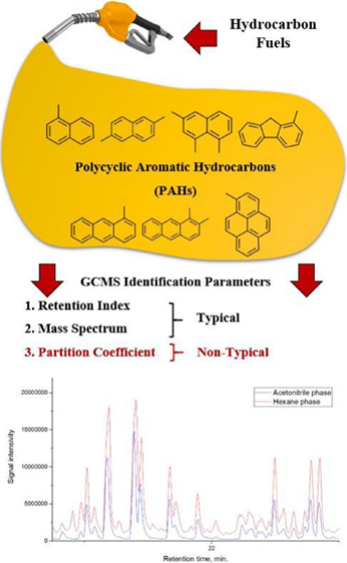

The partition coefficients’ application in the hexane–acetonitrile system as an additional identification feature of polyaromatic hydrocarbons in the review gas chromatography–mass spectrometry analysis of delayed coking gas oils has been considered. The UNIFAC model was used to calculate the partition coefficients of polycyclic aromatic hydrocarbons. It is shown that methyl derivatives of naphthalene, fluorene, anthracene, and pyrene can be identified with an accuracy up to several methyl groups by the position of the figurative point on the partition coefficient–retention index plane. The experimental values of partition coefficients for naphthalene, anthracene, and pyrene are 0.82, 0.78, and 0.77, respectively. The appearance of one methyl group increases the partition coefficient by 0.15–0.2 on average. A total of 53 polyaromatic hydrocarbons were identified in this way.

## 1 Introduction

Analysis of the results given in previous works^1,2^ showed that it would be impossible to replace most hydrocarbon resources with alternatives in the short term. Therefore, the processing of crude oil raw materials will be relevant. The chemical composition of middle distillate oil fractions is their most important parameter, influencing the methods for further processing, storage, and transportation.^3−7^ The components of delayed coking distillate products consist of different classes of hydrocarbon compounds: alkanes, cycloalkanes, alkenes, aromatic hydrocarbons, and various heterocyclic compounds containing oxygen, sulfur, and nitrogen. The hydrocarbon group composition depends on the oil fraction origin. *N*-alkanes mainly represent the straight-run fractions; the term destructive fractions are rich in unsaturated, mono- and polyaromatic compounds. Simultaneously, special attention should be paid to polycyclic aromatic hydrocarbons (PAHs) because the required depth of hydrofinishing reactions in the subsequent processing to obtain environmentally friendly motor fuels depends largely on their quantity.^8−10^ Equally important is the effect of PAHs on the properties of motor fuels.^11−13^ For example, in the case of diesel fuel, PAHs increase the tendency to carbonize and are the main cause of particulate emissions from the diesel engine.^14−16^ PAHs are also highly toxic compounds. The above facts determine the importance of PAH analysis in commercial fuels and in various semi-products of thermodestructive processes.^17−19^

Chromatography is a prerequisite for the analysis of such multicomponent systems. Capillary gas chromatography with FID or MS detection is the most commonly used method for analyzing various petroleum products. When FID detectors are used, peak identification based on the retention index (RI) is sufficient for simple systems. However, such an operation is difficult to be implemented in oil and fuel systems with many isomers and close-boiling components. When MS detectors are used, an additional identification parameter—mass spectrum—appears, enabling the identification of components using mass spectral libraries. However, even the use of MS detectors faces several problems—overlapping peaks and insufficient reliability in identifying some substances. The issue of overlapping peaks seriously complicates the analysis of unsaturated and aromatic hydrocarbons because their peaks often overlap with those of alkanes (the main component of medium distillate hydrocarbon fuels). Besides, the identification of isomeric hydrocarbons is usually impossible using mass spectra because they have very similar spectra that make such identification impractical due to the superposition of the neighboring peaks and noise signals.

Various attempts are being made to improve the quality of analysis of heavy high-boiling oil fractions. The most promising is the application of two-dimensional chromatography (GC × GC). This method of analysis uses two chromatographic columns with different polarities to separate components that do not divide well on a standard nonpolar column. An order of magnitude more peaks can be obtained compared to one-dimensional chromatography. When this method is used to analyze fuel fractions, group identification of compounds can be made quite easily.^20^ This method is also effective for the analysis of nitrogen- and oxygen-containing compounds.^21,22^ The method’s main disadvantage is the need for additional expensive equipment, which significantly increases the analysis complexity and cost. There also remains the problem of substance identification, which requires the knowledge of reference RIs. However, the RIs are significantly dependent on both the column and the temperature program phase. Thus, for anthracene, the linear RI is 1770.90 or 1759.49, depending on the chromatographic column type.^23,24^

Another emerging method of analysis is vacuum ultraviolet spectroscopy.^25^ In this method, the analytical signal is generated by the selective absorption of ultraviolet radiation associated with electronic transitions from the ground state to the excited state. For each compound, the UV spectrum is a specific value that allows identification with great accuracy. An important advantage of this method is the ability to distinguish isomeric hydrocarbons; particularly, it is possible to distinguish isomers with similar RIs, such as 2,6- and 2,7-dimethylnaphthalenes.^26,27^ However, at this point, a significant disadvantage should be mentioned—the absence of databases with hydrocarbon spectra greatly complicates the chromatographic analysis.

Both the abovementioned methods require additional equipment, but it is possible to improve the analytical quality without it. Methods such as gas chromatography with distribution between two phases can be used. The analysis is based on component’s selective extraction, followed by a separate analysis of the two phases. This solves two problems at once. First, by selecting the solvents, the effect of peak overlapping can be reduced; for example, a solvent can be selected that dissolves unsaturated and aromatic compounds well but dissolves alkanes poorly. Second, there is an additional identifying feature, the partition coefficient, which is sensitive to the connection structure. When gas chromatography–mass spectrometry (GC–MS) is used, the researcher has three independent identifying features—retention time, mass spectrum, and partition coefficient. The efficiency of this method has been tested on various models and natural objects.^28,29^ In practice, the most commonly used solvent systems are hexane–acetonitrile and water–octanol. However, the use of the water–octanol system in the case of PAH analysis faces several difficulties. First, the components of fuel systems have extremely low solubility in water, due to which the distribution coefficients of all the components will be large, which can complicate the analysis. Therefore, for toluene, the distribution coefficient is 1.62 × 10^2^.^30^ Second, due to the high viscosity of octanol, problems with phase separation often arise. In the case of acetonitrile, its low viscosity reduces the likelihood of hard-to-separate emulsions. The solubility of the components of fuel systems is high in both acetonitrile and hexane.

The main challenge of such method of analysis is the need to have a partition coefficient database in the chosen solvent system. In simple systems, coefficients can be determined experimentally, but this is practically impossible for the multicomponent hydrocarbon petroleum (fuel) systems because the number of analyzed components is too large. Various semi-empirical and regression models are often used to predict coefficients, but their use requires additional information on substance properties.^31,32^ This raises the question of finding a method for predicting partition coefficients, for which group contribution models of solutions can be used. The most common model is UNIFAC (UNIQUAC Functional-group Activity Coefficients), which is widely used in modeling distillation and extraction in petrochemistry.^33,34^ Within this model’s framework, it is possible to calculate partition coefficients for hydrocarbons of any composition.^35^

This work’s main objective is to develop a working methodology for the chromatography distribution method application in combination with model calculations for the identification of polyaromatic hydrocarbons of delayed coking products.

## 2 Experimental Section

### 2.1 Tools and Materials

Chromatographic measurements were performed on a Shimadzu GC-QP2010SE chromatography–mass spectrometer (Shimadzu Scientific Instruments, Inc., Columbia MD) equipped with an RTX-5MS (30 m × 0.25 mm × 0.25 μm) column from Restek. The chromatograph was operated in a constant velocity mode (1.03 mL/min). The carrier gas was helium. The evaporator temperature was 250 °C. The injected sample volume was 1 μL and the split ratio was 20:1. The furnace program consisted of an initial isotherm of 50 °C (for 10 min), then the temperature was increased to 290 °C at a rate of 10 os/min and held for 10 min. The ion-source temperature of the mass detector was 200 °C and the interface temperature was 290 °C. The mass-scanning range was chosen as 45–500 *m*/*z* with an acquisition time of 0.3 s.

Samples of commercial diesel fuel and delayed coking gas oils were used as test objects. The solvents were acetonitrile (for HPLC) and hexane (for HPLC).

### 2.2 Preparation of Equilibrium Two-Phase Systems for GC–MS Analysis

The sample preparation consisted of two sequential extraction processes. In the first step, 1 mL of the test mixture was placed in a 10 mL glass vial with a silicone stopper, then 2 mL of acetonitrile was added and shaken vigorously for 5 min. The sample was then left to rest for 10 min for delamination and 1 mL of the acetonitrile phase was taken. The acetonitrile phase was placed on top, in the case of diesel fuel and delayed coking gas oils. The selected acetonitrile phase was transferred into a new 10 mL glass vial and 2 mL of hexane was added. The mixture was again shaken vigorously for 5 min and allowed to dissolve for 10 min. Extraction was carried out at 20 °C. This variant of sample preparation distorts the initial composition of the analyzed mixture, namely, enriches it with unsaturated and aromatic compounds and impoverishes it with *n*-alkanes. Because aromatic compounds are not the main components of diesel fuel, this method can be considered as a method of PAH preconcentration.

### 2.3 Chromatogram Layout and RI Calculations

All chromatograms were processed according to a standard Shimadzu GC–MS solution method program, which took into account the baseline drift, with a determined maximum peak width (3 s) and a minimum peak area. A standard report with the output time and area of each peak was generated by the results of processing. The linear Kovacs RIs for the sample components were calculated using the TIC (total ion chromatogram) because all the samples studied contained *n*-alkanes with C_11_–C_25_ carbon atoms. Undecane, tridecane, pentadecane, heptadecane, nonadecane, heneicosane, and pentacosan were selected as the defining alkanes. The average scatter of the yield times of the defining alkanes did not exceed 0.02 min. Accordingly, the scatter of the calculated RIs could not exceed 2 units, except for substances coming after C_21_, for which it could not exceed 4 units. The identification of the compounds was done by the combined use of mass spectra and RI. Library mass spectra and linear RIs were taken from the NIST 11 database. A substance was considered identified if its mass spectrum corresponded to the library one by 70%, the acceptable variation of RIs was ±30 units. The integration of the peaks was performed by both TIC and SIM (selected ion monitoring).

### 2.4 Determination of Partition Coefficients

This paper uses three types of partition coefficients—calculated, experimental, and corrected. The UNIFAC model was used for the estimated partition coefficient. Under conditions of thermodynamic equilibrium, for each component in the case of two limitedly soluble liquids, the equality is fulfilled as follows
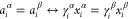
1where *a*_*i*_^α^ and *a*_*i*_^β^ are the activities of the *i*-th component in phases α and β, respectively; *x*_*i*_^α^ and *x*_*i*_^β^ are the mole fractions of the component; and γ_*i*_^α^ and γ_*i*_^β^ are the activity coefficients of the *i*-th component.

Because the concentrations of the studied polyaromatic hydrocarbons are small, both phases can be considered as diluted solutions for these components. In such solutions, the activity coefficients cease to depend on concentrations. Thus, the expression is valid
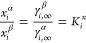
2where γ_*i*,∞_^α^ and γ_*i*,∞_^β^ are the limiting activity coefficients of the *i*-th component in phases α and β, respectively, and *K*_*i*_^x^ is the partition constant, expressed in terms of mole fractions.

The following equation defines the relationship between the concentration coefficient and the distribution constant:
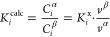
3where *K*_*i*_^calc^ is the concentration coefficient; *C*_*i*_^α^ and *C*_*i*_^β^ are the concentration of the *i*-th component in phases α and β, respectively, and *v*^α^ and *v*^β^ are the molar volumes of the coexisting phases α and β, respectively.

The activity coefficient values of components were calculated within the framework of the UNIFAC model. It divides the substance into separate groups, which are characterized by their volume and surface area. Also, the parameters of groups interacting with each other—*a*_*ij*_ and *a*_*ji—*_are introduced, which show the degree of difference in the interaction of groups *i* and *j* in comparison with interactions *i*–*i* and *j*–*j*. The logarithm of the activity coefficient is represented as the sum of two summands
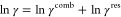
4

The first term is called the combinatorial component and the second term is called the residual component. The combinatorial component depends only on the composition of the solution and the volume and surface area of the constituent groups. The Staverman–Guggenheim equation is used to calculate the combinatorial component, as in the UNIQUAC model 35
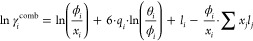
5where *x*_*i*_ is the mole fraction of the component; ϕ_*i*_ and θ_*i*_ are the volume and surface fraction of the component, respectively; and *l*_*j*_ is the bulk factor of the molecule.

The residual part of the activity coefficient includes the energy interactions of the groups. This part is represented by the sum of the components of the individual groups that make up the molecule
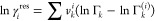
6where Γ_*k*_ and Γ_*k*_^(*i*)^ are the residual activity coefficients of group *k* in the test solution and in a pure liquid consisting only of substance *i*, respectively.

The equilibrium composition calculation of the two phases was also based on the condition of equality of the component activities in the two phases. This is done by varying the amount of substance (ξ_*i*_), which has passed from phase 1 to phase 2, according to the following equation
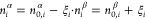
7where *n*_*i*_^α^ and *n*_*i*_^β^ are the amount of the *i*-th substance in the phases α and β, respectively, and *n*_0,*i*_^α^ and *n*_0,*i*_^β^ are the initial amount of the *i*-th substance in phases α and β, respectively.

The next function was minimized as follows
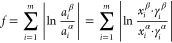
8

Since in equilibrium the activities of an individual component in two phases are equal, the logarithm of the ratio *a*_*i*_^β^/*a*_*i*_^α^ will tend to 0. Therefore, the optimized function will tend to 0 under conditions close to equilibrium. A binary search algorithm combined with a greedy approach was used to find the optimal composition. For this, the reaction (in the form of eq 7) with the greatest deviation from the equilibrium was chosen, which was brought into equilibrium independent of the others. The search for the equilibrium was carried out by dividing the segment of possible values ξ_*i*_ by 2. For example, if the component is present in only one phase, then for the first iteration ξ_*i*_ = *n*_0,*i*_/2. For subsequent iterations, the direction of change ξ_*i*_ depended on the value of the ratio *a*_*i*_^β^/*a*_*i*_^α^. If the ratio is greater than 0, then the value of ξ_*i*_ decreased, if it is less than 0, it increased. If at the second iteration, the ratio is less than 0, then , and so on until equilibrium is reached. The composition was considered to be in equilibrium (eq 8) at the value *f* = 10^–4^. The calculation was performed using a package written in the python programing language.^36^

The experimental values of partition coefficients were determined by the chromatographic method. Traditionally, it is assumed that the concentration of a sample component is proportional to the chromatographic peak area of the component
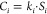
9where *C*_*i*_ is the concentration of the component in the sample; *k*_*i*_ is the sensitivity coefficient for the *i*-th substance; and *S*_*i*_ is the area of the chromatographic peak.

Using this expression, we can find the experimental partition coefficient as
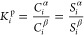
10where *S*_*i*_^α^ and *S*_*i*_^β^ are the area of the chromatographic peak of the *i*-th component in phases α and β, respectively.

Such ratios are usually calculated using an internal standard. Any substance with a known partition coefficient is taken as such. This method of correction takes into account input volume errors and is optimal when it is necessary to compare the measured partition coefficients with those already known. In the case of polyaromatic hydrocarbons, the partition coefficients are unknown. For this reason, the calculated values of partition coefficients for naphthalene, 1-methylnaphthalene, anthracene, and pyrene were used as internal standard. As a result, the following linear equation was used to correct the experimental partition coefficient
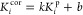
11where *K*_*i*_^cor^ is the corrected partition coefficient and *k* and *b* are parameters determined using the linear regression.

## 3 Results and Discussion

### 3.1 Calculation of the Equilibrium Composition and Partition Coefficients of Some Classes of Organic Compounds

To calculate the partition coefficients in two-phase systems, it is necessary to know the two immiscible phases’ equilibrium compositions. The phase composition has a significant influence on the activity coefficient of a single component. The equilibrium composition of the phases in the hexane–acetonitrile system was repeatedly determined by the experiment and calculation. The main challenge is that petroleum products contain many individual components, which greatly complicate the calculation. However, in terms of group solution models, the main components of petroleum products (alkanes, arenes, and cycloalkanes) can be represented as a set of only three groups—CH_3_ (alkyl), ACH (aromatic), and ACCH_3_ (hydrocarbon radical at the aromatic cycle). Thus, the two immiscible phases—hexane and acetonitrile—are represented by four groups—CH_3_, ACH, ACCH_3_, and CH_3_CN.

To study the influence of the petroleum product chemical composition (primarily the amount of aromatic compounds) on the partition coefficients of the individual component in the extraction process, this system was presented as a mixture of hexane, benzene, and acetonitrile. This choice of substances is because hexane and acetonitrile are direct participants of extraction equilibrium and benzene is a component with aromatic groups. The first phase was a mixture of hexane and benzene in different proportions, a model petroleum product (a mixture of CH_3_ and ACH groups). Pure acetonitrile served as the second phase, and toluene was chosen as the test substance.

Table 1 shows the calculated equilibrium compositions of the two phases (components’ molar fractions) in the hexane–benzene–acetonitrile system and the value of the toluene partition coefficient in these systems. The compositions of phases received as a calculation result agree well with the experimental values from which it is possible to conclude the received values’ reliability.^37^ As can be seen, the aromatic compound content in the initial sample has little effect on toluene’s partition coefficient. An increase in the number of aromatic compounds in the system increases acetonitrile solubility in hexane and hexane in acetonitrile. Because the matrix’s influence on the partition coefficient is insignificant for further calculations, the phase compositions obtained in calculation number 1 will be used.

**Table 1:** Results of the Equilibrium Composition Calculation of the Hexane–Benzene–Acetonitrile System

no.	*x*_hexane_^α^	*x*_benzene_^α^	*x*_acetonitrile_^α^	*x*_hexane_^β^	*x*_benzene_^β^	*x*_acetonitrile_^β^	*K*_toluene_^calc^
1	0.9360	0.0065	0.0576	0.0655	0.0036	0.9309	0.96
2	0.9048	0.0322	0.0629	0.0683	0.0182	0.9135	0.96
3	0.8654	0.0642	0.0703	0.0720	0.0371	0.8909	0.97
4	0.7847	0.1275	0.0879	0.0809	0.0772	0.8419	0.97
5	0.7005	0.1893	0.1102	0.0924	0.1211	0.7865	0.98
6	0.6114	0.2488	0.1398	0.1079	0.1699	0.7222	0.98

Table 2 presents the partition coefficients’ calculation results (acetonitrile/hexane) for the representatives of different classes of hydrocarbons and heterocyclic compounds at the specified compositions of equilibrium phases.

**Table 2:** Calculated Partition Coefficients of Various Substances in the Hexane–Benzene–Acetonitrile System

molecule	*K*_*i*_^calc^	group composition of the molecule	estimated nonpolar RI
octane	9.54	2·CH_3_ 6·CH_2_	800
methylheptane	9.52	3·CH_3_ 4·CH_2_ 1·CH	752
2,2,4-trimethylpentane	9.86	5·CH_3_ 1·CH_2_ 1·CH 1·C	668
tridecane	33.40	2·CH_3_ 11·CH_2_	1300
pentadecane	55.13	2·CH_3_ 13·CH_2_	1500
heptadecane	91.00	2·CH_3_ 15·CH_2_	1700
cyclohexane	4.34	6·CH_2_	719
methylcyclohexane	5.58	1·CH_3_ 5·CH_2_ 1·CH	781
naphthalene	0.78	8·ACH 2·AC	1231
methylnaphthalene	0.91	7·ACH 2·AC 1·ACCH_3_	1345
dimethylnaphthalene	1.05	6·ACH 2·AC 2·ACCH_3_	1458
trimethylnaphthalene	1.21	5·ACH 2·AC 3·ACCH_3_	1571
methylethylnaphthalene	1.50	6·ACH 2·AC 1·ACCH_2_ 1·CH_3_ 1·ACCH_3_	1571
fluorene	0.76	9·ACH 4·AC	1494
methylfluorene	0.87	8·ACH 4·AC 1·ACCH_3_	1607
anthracene	0.74	10·ACH 4·AC	1782
methylanthracene	0.85	9·ACH 4·AC 1·ACCH_3_	1896
dimethylanthracene	0.99	8·ACH 4·AC 2·ACCH_3_	2009
pyrene	0.73	10·ACH 6·AC	1984
methylpyrene	0.84	9·ACH 6·AC 1·ACCH_3_	2097
tetracene	0.69	12·ACH 6·AC	2210
pyridine	0.14	1·C_5_H_5_N	674
methylpyridine	0.26	1·CH_3_ 1·C_5_H_4_N	787

RIs were calculated according to the additive scheme.^38^ As can be seen, PAHs are distributed between the two phases with partition coefficients close to unity (0.78–0.69). As the number of aromatic cycles increases, the solubility of PAHs in the acetonitrile phase slowly increases. The addition of hydrocarbon radicals to the PCA molecule increases the solubility in the hexane phase, and the radical attached directly to the aromatic ring has a smaller influence (partition coefficient of dimethylnaphthalene is 1.05 and that of ethylnaphthalene is 1.29) at that. In the *n*-alkane series, the equilibrium is significantly shifted in the hexane phase direction, hence with the increase of the length of the hydrocarbon chain the partition coefficient grows rapidly. The alkane structural isomerism affects the component distribution in this system insignificantly, so for *n*-octane, the coefficient is 9.54 and for methylheptane it is 9.52, which in terms of practical determination of coefficients is an insignificant deviation. Nitrogen-containing heteroatomic compounds are much better soluble in the acetonitrile phase, which is confirmed by the example of pyridine and methylpyridine.

**Figure 1:**
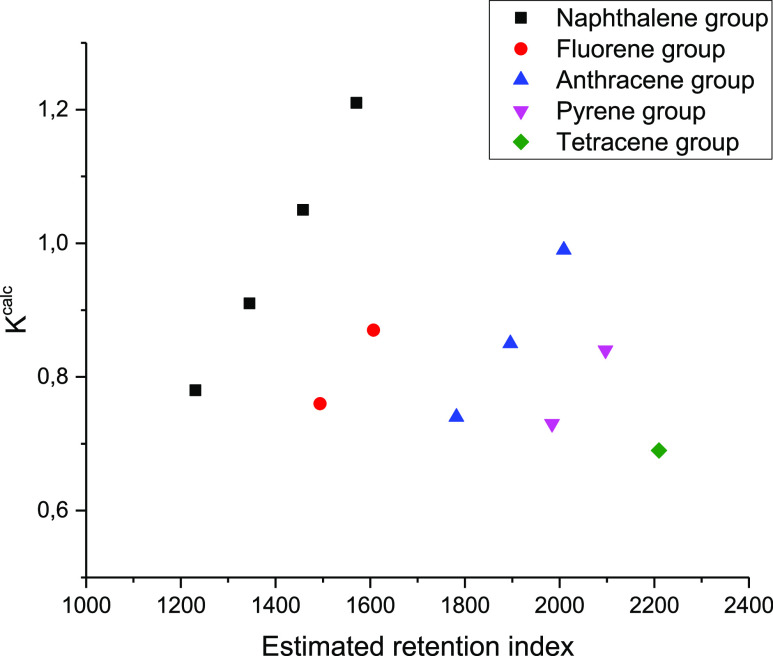
Figurative points’ location of representatives of different homologous series of PAHs on the EST RI plane and the calculated partition coefficient.

Thus, based on the calculations of the model UNIFAC, we can conclude that the system hexane–acetonitrile is suitable for the study of polycyclic hydrocarbons, as their partition coefficients are sufficiently close to 1, which will provide the determined concentrations of the components in both phases. Also, the partition coefficient is quite sensitive to the number of methyl radicals (each radical will change the coefficient by 0.15–0.20 units). The combined use of the estimated RI (EST RI) and the estimated partition coefficient *K* will provide two identifiers using only the knowledge of the substance composition; an example of this approach is shown in Figure 1. As can be seen, all the PAHs presented differ in either RIs or partition coefficients. Given the mass spectra, this allows their unambiguous identification.

### 3.2 Determination of Naphthalene, Anthracene, and Pyrene Partition Coefficients

In order to confirm the agreement of the calculated partition coefficients with the practical ones, the gas chromatography determination of *K* for naphthalene, anthracene, and pyrene in the hexane–acetonitrile system was performed. The choice of substances is driven by several factors. First, these substances are the simplest representatives of the corresponding homologous series. Second, naphthalene, anthracene, and pyrene are often used to create the Lee RI scale, which accounts for their prevalence in petrochemical laboratories.^39^ For each substance, the number of repeated measurements was at least 5. The results are recorded in Table 3.

**Table 3:** Partition Coefficients of Reference Substances in Model and Real Systems

molecule	linear RI	estimated nonpolar RI	*K*_et,*i*_^p^, average value	*K*_*i*_^p^	*K*_*i*_^calc^
naphthalene	1190.8 ± 0.4	1231	0.82 ± 0.08	0.82	0.78
anthracene	1820.8 ± 0.4	1782	0.78 ± 0.08	0.69	0.74
pyrene	2164.8 ± 0.4	1984	0.77 ± 0.08	0.63	0.73

The partition coefficients determined chromatographically agree well with those calculated from the UNIFAC model. The relative discrepancy between the calculated experimental values does not exceed 10%, while the error of experimental determination is 10%.

To estimate possible distortions at the partition coefficient calculation of components in chromatograms of real hydrocarbon mixtures, partition coefficients of naphthalene, anthracene, and pyrene were determined according to the results of the real chromatogram processing of delayed coking light gas oil (*K*_*i*_^p^ from Table 3). As can be seen, these coefficients differ both from those determined on the reference samples and from those calculated by the UNIFAC model. This fact may be due to several reasons. First, the stability of the sample introduced into the chromatograph has significant influence on the value of *K*, because for the practical value to exactly correspond with the tabulated value the introduced volume for the two phases must be identical. Second, the *K* determination is affected by the peak overlap. This is due to the inaccuracy of the peak area calculation in the automatic integration mode. In the case of the MS detector, chromatograms taken in the TIC mode are primarily affected. Most often, this problem can be solved using integration by the characteristic ion rather than by TIC. Also, in such calculations, it is necessary to take into account the imperfections of group models.

Comparing the last three columns of Table 3, we see that the discrepancy between the partition coefficients determined in the real and model systems and those calculated by the UNIFAC model does not exceed 20%.

### 3.3 Identification of PAHs in Real Mixtures

**Figure 2:**
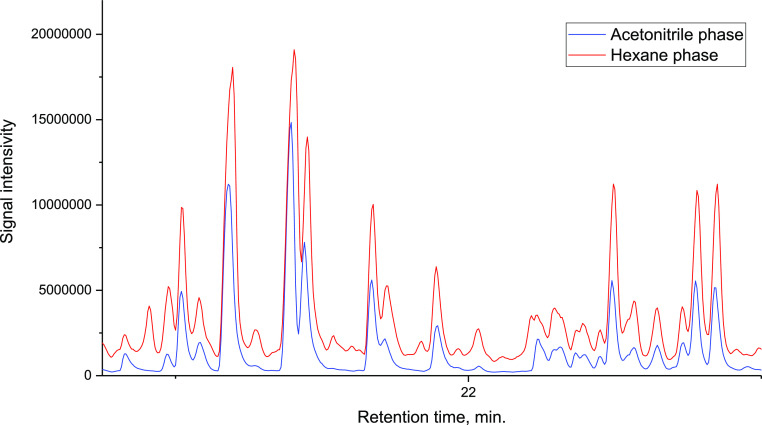
Chromatogram section of acetonitrile and hexane phases of the diesel fuel extract.

Figure 2 shows the same time sections of chromatograms of acetonitrile and hexane phases in the yield region of methyl- and dimethylnaphthalenes. It can be seen that the hexane phase is richer in components compared to the acetonitrile phase. This is characteristic for all areas of the chromatogram on the whole; for this reason, the number of identified components in the hexane phase is 224 and in the acetonitrile phase it is 181; the partial overlapping of peaks makes it difficult to identify components in the hexane phase and determine the area of the peaks. All peaks absent in the acetonitrile phase but present in the hexane phase refer to alkane hydrocarbons. For this reason, all partition coefficients were calculated from the peak areas of the characteristic ions. Thus, for atracene, naphthalene and pyrene molecular ions were used.

In the present work, a correlation equation linking the values of partition coefficients of naphthalene, methylnaphthalene, anthracene, and pyrene calculated for this pair of chromatograms with the similar values calculated by UNIFAC is used. In this way, the effect of the inequality of acetonitrile volumes and hexane phases during chromatography on the value of the partition coefficient is taken into account. Such a correction also allows taking into account systematic deviations of the partition coefficients’ calculated values from the actual values. Methylnaphthalene was selected as an additional substance to naphthalene, atracene, and pyrene. The choice is due to the fact that this substance has only two isomers, α- and β-methylnaphthalene, which means that it is easy to detect in the chromatogram. The introduction of a methyl-substituted PAH derivative improves the accuracy of the correction in the high-value region.

**Figure 3:**
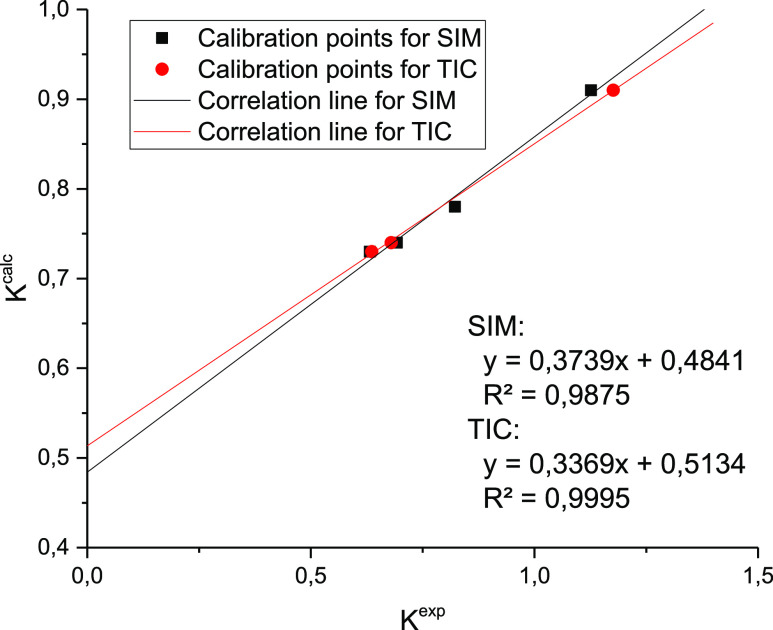
Calibration points for SIM and TIC chromatograms.

Figure 3 depicts the calibration points for the delayed coking gas oil sample; as can be observed for SIM and TIC chromatograms, there is a significant correlation in the coordinates calculated partition coefficient and experimental partition coefficient. However, in the TIC chromatogram, it was not possible to determine the experimental partition coefficient for naphthalene due to overlapping peaks. Thus, a similar linear correction will correct observables at a single measurement toward agreement with the calculated values for all the investigated compounds.

**Figure 4:**
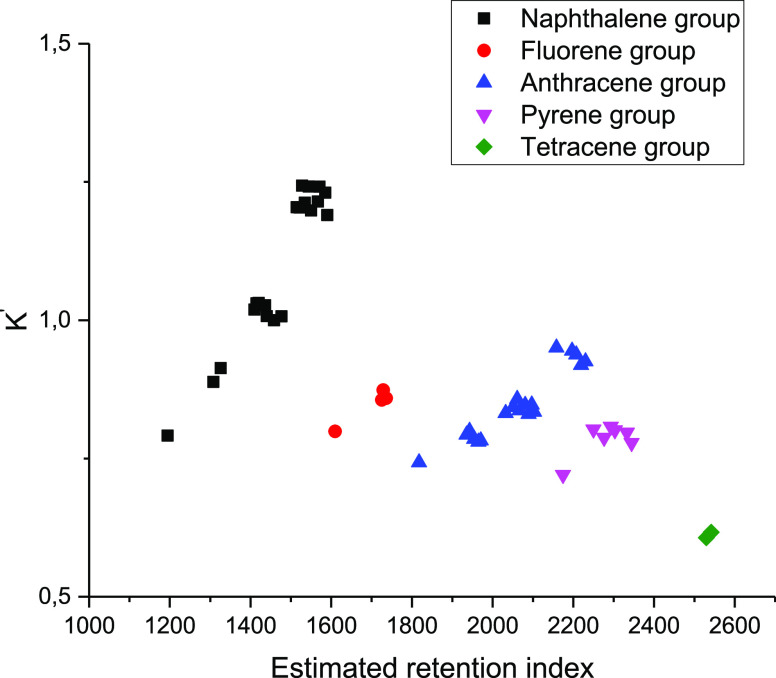
Compounds’ figurative point location (integration of the peak was carried out on the characteristic ion).

Figure 4 shows the figurative points of both identified and potential PAHs in the coordinates’ linear RI–corrected partition coefficient. Because UNIFAC is insensitive to the mutual arrangement of aromatic rings and the position of methyl substituents, identification is possible only to the molecule’s exact group composition. Three groups of compounds—methylnaphthalenes, dimethylnaphthalenes, and trimethylnaphthalenes—were chosen for naphthalene derivatives. Of the fluorene derivatives, the methylfluorene group was selected. In the case of anthracene derivatives, identification occurred in the series of compounds methylanthracene–methylphenanthrene, dimethylanthracene–dimethylphenanthrene, and trimethylanthracene–trimethylphenanthrene. This separation is caused by the fact that phenanthrene and anthracene have the same group-partitioning and hence the same calculated partition coefficients in terms of group solution models. Substances containing four benzene rings were chosen as the last set of compounds.

As can be observed for the figurative points obtained from the characteristic ions, a noticeable clustering is observed. The experimental points’ location is similar to the location in the model coordinates of the corrected partition coefficient–calculated RI, which indicates the good predictive ability of the model for such calculations. As the number of substituents in the aromatic cycle increases, the figurative points move away from the origin for each corresponding series, which corresponds to an increase in the equilibrium concentration in the hexane phase. Within the same homologous series, there is a good separation of the corrected partition coefficient *K*′ within the same group of compounds, which is best shown by the example of naphthalene homologues. Within one section of the chromatogram, only substances belonging to one group are released with a certain separation factor. Thus, for methylnaphthalene, methylfluorene, dimethylanthracene, and methylpyrene, which have *K* of the order of 0.9, the difference in RIs is 350–400 units. This fact allows the partition coefficient to be used without problems as an additional identifying feature in GC–MS analysis.

Table 4 shows peak parameters of the trimethylnaphthalene group. For each peak, the real mass spectrum correspondence with the library one was more than 95% (trimethylnaphthalene/methylethylnaphthalene has a very similar mass spectrum). However, in 4 cases out of 10, the automatic identification program proposed erroneous variants, if trimethylnaphthalene/methylethylnaphthalene is taken for correct identification. This is because the actual RIs of substances are outside the limits specified in the program. In this case, the application of partition coefficients allows not only to determine whether the substance belongs to substituted naphthalenes but also to distinguish trimethylnaphthalene from methylethylnaphthalene. The practical partition coefficients turn out to be grossly overestimated. This is primarily due to the fact that the volume of the introduced hexane phase was greater than the volume of the acetonitrile phase. By adjusting with internal standards (Figure 3), values close to the calculated ones can be obtained. The mean value of the corrected partition coefficient, except for substances with RIs 1528 and 1590, is 1.22, while the calculated one is 1.21. In peaks with *K* equal to 1.51 and 1.54, such values can be interpreted as methylethylnaphthalene (calculated partition coefficient is 1.5).

**Table 4:** Comparative Results of Identifying a Group of Compounds with RIs of 1514–1590 According to the Mass Spectrum–Distribution Ratio and Mass Spectrum–RI Criteria (Trimethylnaphthalene Distribution Ratio 1.21 and Methylethylnaphthalene 1.5, EST RI 1571)

linear RI	*K*_p_′	identification by mass spectrum and partition coefficient	the result of automatic identification by mass spectrum (>70%) and RI (±30)
1514	1.20	trimethylnaphthalene	not identified
1521	1.20	trimethylnaphthalene	5,9-methano-5*H*-benzocycloheptene, 8-bromo-8,9-dihydro-
1528	**1.54**	methylethylnaphthalene	5,9-methano-5*H*-benzocycloheptene, 8-bromo-8,9-dihydro-
1535	1.21	trimethylnaphthalene	5,9-methano-5*H*-benzocycloheptene, 8-bromo-8,9-dihydro-
1544	1.24	trimethylnaphthalene	naphthalene, 2,3,6-trimethyl-
1550	1.19	trimethylnaphthalene	naphthalene, 2,3,6-trimethyl-
1567	1.21	trimethylnaphthalene	naphthalene, 2,3,6-trimethyl-
1571	1.24	trimethylnaphthalene	naphthalene, 2,3,6-trimethyl-
1585	1.23	trimethylnaphthalene	naphthalene, 2,3,6-trimethyl-
1590	**1.51**	methylethylnaphthalene	naphthalene, 2,3,6-trimethyl-

Table 5 presents the peak parameters of the methylatracene/methylphenanthrene group. For each peak, the correspondence of the real mass spectrum with the library one was more than 92%. As can be seen in this case, the automatic identification by mass spectra and RIs did not allow the compounds to be recognized. This is because these compounds’ RIs differ by more than 30 units for an established chromatographic column.

**Table 5:** Comparative Results of Identifying a Group of Compounds with RIs of 1935–1970 by Mass Spectrum–Partition Coefficient and Mass Spectrum–RI Criteria (Methylanthracene Partition Coefficient 0.85, EST RI 1896)

linear RI	*K*_*i*_^cor^	identification by mass spectrum and partition coefficient	the result of automatic identification by mass spectrum (>70%) and RI (±30)
1935	0.81	methylanthracene\methylphenanthrene	cyclopropene, 3-bromo-1,2-diphenyl-
1943	0.80	methylanthracene\methylphenanthrene	cyclopropene, 3-bromo-1,2-diphenyl-
1955	0.83	methylanthracene\methylphenanthrene	[1,1′-biphenyl]-4-carbonitrile, 4′-propyl-
1965	0.80	methylanthracene\methylphenanthrene	(1,1′-biphenyl)-2,2′-dicarboxaldehyde
1971	0.80	methylanthracene\methylphenanthrene	[1,1′-biphenyl]-4-carbonitrile, 4′-propyl-

**Figure 5:**
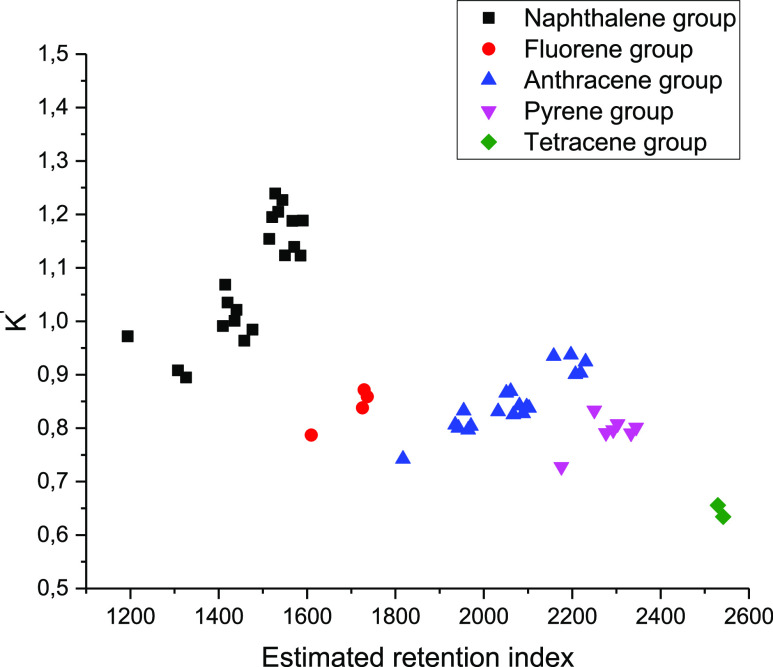
Location of figurative connection points. Iteration of the peaks was performed by TIC.

As shown in Figure 5, the scatter of the partition coefficients increases when the TIC chromatogram is used. This is primarily due to the difficulty in accurately calculating the peak area, as the imposition or overlap often occurs in fuel systems. However, even in this case, the characteristic clustering for component groups persists. For the given chromatogram, the value of the partition coefficient of one of the reference substances (naphthalene) was distorted due to the superposition of two peaks; naphthalenelene was excluded from the correlation equation while calculating the corrected coefficient. The corrected coefficients are also close to the calculated values, but the identification between some groups can be difficult due to significant variation.

Table 6 presents the results of polyaromatic hydrocarbon identification using the described methodology.

**Table 6:** Summary Table of the Identification Results of Semi-aromatic Hydrocarbons by Mass Spectra and Partition Coefficients

group of compounds	characteristic ion, *m*/*z*	RI range	*K*_*i*_^calc^	*K*_*i*_^cor^ by characteristic ion	*K*_*i*_^cor^ by TIC
methylnaphthalenes	142	1308–1326	0.91	0.90	0.89
dimethylnaphthalenes	141	1458–1477	1.05	1.02	1.00
methylethylnaphthalene	155	1528–1590	1.50	1.53	1.31
trimethylnaphthalenes	155	1514–1590	1.21	1.22	1.17
methylfluorenes	165	1726–1736	0.87	0.86	0.85
methylanthracenes	192	1935–1971	0.85	0.79	0.80
dimethylanthracenes	206	2032–2103	0.99	0.85	0.84
trimethylanthracenes	220	2158–2230	1.14	0.94	0.92
methylpyrenes	216	2250–2345	0.84	0.80	0.80

As might be expected, the dispersion of the RIs increases with the number of methyl substituents. This makes automatic reliable identification of such compounds without regard to the partition coefficient much more difficult. Comparing the partition coefficients by characteristic ions and by TIC, it can be noted that the use of characteristic ions is not strictly obligatory. The coefficients obtained in both ways have values close to each other. The standard deviation of the values for the characteristic ion coefficients was 0.01 and that of the TIC chromatogram was 0.04. This means that the TIC values should be treated with caution as they are strongly influenced by both chromatogram’s quality (overlapping and imposition) and the quality of automatic peak labeling and integration.

## 4 Conclusions

In general, the use of partition coefficients makes it possible to avoid false identifications based on the lack of actual data on PAH RIs, on the one hand, and on the inevitable scatter of the chromatographic column properties, on the other hand. It is fundamentally important to have an adequate model (empirical or thermodynamic) to calculate the partition coefficients because the databases on such quantities are not large enough. Further refinement of PAH identification attributes requires either applying known standards or selective derivatization methods, which is hardly feasible in the framework of in-line analysis of the diesel fraction composition and products of thermally destructive processes, in particular, delayed coking gas oils. It is important to understand that RIs are not completely eliminated from the analysis because there is a clustering of polyaromatic compounds with a single number of methyl groups on the RI–distribution factor plane. The sequence of compounds’ output times within the same class is also preserved. It is also possible to use this method with a PID detector, which will give a second identification parameter for each peak in the chromatogram. An equally important advantage is the purification of the chromatogram from alkanes and cycloalkanes, which also greatly simplifies identifying the components of the oil fractions.
